# A Screen for Retrotransposed Imprinted Genes Reveals an Association between X Chromosome Homology and Maternal Germ-Line Methylation

**DOI:** 10.1371/journal.pgen.0030020

**Published:** 2007-02-09

**Authors:** Andrew J Wood, Roland G Roberts, David Monk, Gudrun E Moore, Reiner Schulz, Rebecca J Oakey

**Affiliations:** 1 Department of Medical and Molecular Genetics, King's College London, London, United Kingdom; 2 Unit of Clinical and Molecular Genetics, Institute of Child Health, London, United Kingdom; University of Cambridge, United Kingdom

## Abstract

Imprinted genes undergo epigenetic modifications during gametogenesis, which lead to transcriptional silencing of either the maternally or the paternally derived allele in the subsequent generation. Previous work has suggested an association between imprinting and the products of retrotransposition, but the nature of this link is not well defined. In the mouse, three imprinted genes have been described that originated by retrotransposition and overlap CpG islands which undergo methylation during oogenesis. *Nap1l5, U2af1-rs1,* and *Inpp5f_v2* are likely to encode proteins and share two additional genetic properties: they are located within introns of host transcripts and are derived from parental genes on the X chromosome. Using these sequence features alone, we identified *Mcts2,* a novel candidate imprinted retrogene on mouse Chromosome 2. *Mcts2* has been validated as imprinted by demonstrating that it is paternally expressed and undergoes promoter methylation during oogenesis. The orthologous human retrogenes *NAP1L5, INPP5F_V2,* and *MCTS2* are also shown to be paternally expressed, thus delineating novel imprinted loci on human Chromosomes 4, 10, and 20. The striking correlation between imprinting and X chromosome provenance suggests that retrotransposed elements with homology to the X chromosome can be selectively targeted for methylation during mammalian oogenesis.

## Author Summary

The conventional view is that DNA carries all of our heritable information and our genes control development into adulthood. The discovery of epigenetics, a term coined to describe effects that are not coded for by DNA sequence, but can nonetheless affect our development and well-being, has added another layer of complexity to our understanding of genetics. One class of genes under epigenetic control are imprinted genes. Mammals inherit two copies of every gene, one from mother and one from father, and in most cases, both are active. However, for a small number of imprinted genes in mammals, only one is active, either the maternal or the paternal copy. Epigenetics amounts to a control system for switching genes on and off appropriately. We focus on a group of little-studied imprinted genes that share features that give clues to their evolutionary origins. These so-called “retrogenes” are protein-coding sequences of DNA that have undergone duplication and jumped into novel locations in the genome. Because of this, it is possible to determine where, and roughly when, many of the imprinted retrogenes originated. This provides an opportunity to study the molecular events that have generated imprinted genes during mammalian evolution.

## Introduction

Mammals inherit one haploid genome complement from each parent, and in most cases both alleles are expressed and functionally equivalent. Imprinted alleles are an exception to this rule, as their expression in offspring is dependent on the gender of the transmitting parent. These parent-of-origin effects arise due to differential epigenetic reprogramming events occurring in the male and female germ-line. Methylation at CpG dinucleotides is one modification known to play a key role, and germ-line differentially methylated regions (gDMRs) have been found in proximity to most known imprinted genes. In addition to performing an essential role in genomic imprinting [1], DNA methylation also serves to suppress the activity of retrotransposon promoters [2,3]. This connection led to the proposal that the two processes may be mechanistically linked [4–7], which is further supported by the identification of imprinted genes with retrotransposon-like properties [8].

Following the wealth of sequence data that has been made available in recent years, the conceptual distinction between genes and transposons has become increasingly vague. For example, autonomously replicating L1 retroelements can be diverted to act on host cell mRNAs [9], suggesting that almost any cellular mRNA has the capacity to act as a retrotransposon. A recent survey identified 3,590 of these intronless gene duplicates in the human genome, of which 1,080 showed evidence of transcription [10]. More than 100 have maintained the capacity to encode proteins, indicating that retrotransposition is a major source of protein-coding novelty in mammals [10]. We adopt the term “retrogene” hereafter to refer to these putatively functional elements [10–12], as distinct from the genetically disabled “retropseudogenes.”

Due to the mechanistic link discussed above, it is not surprising that a small number of retrogenes have been shown to undergo imprinting [13,14]. One such gene, murine *U2af1-rs1,* is a retrotransposed copy of the X-linked *U2af1-rs2* gene, which lies within an intron of *Murr1* on Chromosome 11 [13]. The orthologous human locus lacks the retroposed sequence and a differentially methylated CpG island [15], indicating that the gene duplication occurred after the divergence of rodents and primates (∼65 million years ago). The human *MURR1* gene shows no evidence of imprinted expression or allele-specific methylation, indicating that imprinting at this locus arose at about the same time point in rodent evolution as the retroposon insertion [15].

To investigate the link between retrotransposition and genomic imprinting further, we performed a systematic screen of known imprinted genes in the mouse to identify candidate retrogenes. Eleven genes were identified, three of which have CpG islands overlapping the retrotransposed exons that undergo differential germ-line methylation. The other eight are likely to be controlled by differentially methylated elements that are not within the duplicated sequences. The three retrogenes share three sequence characteristics, namely, they are located within an intron of another gene, they are derived from an ancestral gene on the X chromosome, and they are associated with an overlapping CpG island. These characteristics alone were used to identify a novel imprinted locus consisting of *Mcts2* and *H13,* a pair of reciprocally expressed novel imprinted genes on mouse Chromosome 2. Finally, we show that imprinting is conserved in humans for the three retrogenes that predate the divergence of rodents and primates.

## Results

A database of known imprinted genes in the mouse is housed on the Harwell imprinting Web site [16] and the 76 currently listed were screened to identify candidate retrogenes (Dataset S1 and Text S1). Protein-coding capacity was ascertained from the references linked to each gene entry in the same database [16]. To identify imprinted genes likely to have been associated with sequence duplications, the BLASTZ tool [17] (integrated into the self-chain track on the University of California, Santa Cruz genome browser, Mm build 34) was utilized to identify those that generated alignments with regions elsewhere in the mouse genome. The Harwell database includes a significant proportion of genes for which no functional open reading frame (ORF) has been identified, many of which are known to act as noncoding RNAs. Of the 55 for which a putatively functional ORF has been identified, 41 (75%) generated BLASTZ self-alignments and are likely to be either the source or product of sequence duplications. To enrich for genes likely to have originated via an mRNA intermediate, genes with introns in their ORF were excluded. The remaining 11 represent candidate retrogenes (Table 1), although the three that lack multi-exonic paralogues cannot be definitively classified as such. It should be noted that *Rtl1* and *Peg10* belong to a family of endogenous retroviral elements that have lost the capacity to replicate in an autonomous manner [8], and hence may be considered distinct to the remaining nine genes that show no evidence of retroviral homology.

**Table 1:**
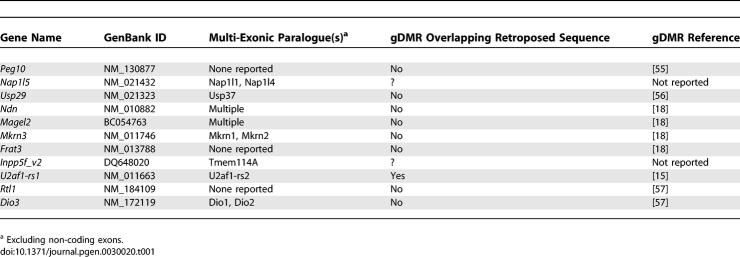
Murine Imprinted Genes Exhibiting Characteristic Features of Retrotransposition

All 11 candidates are expressed from the paternally derived allele. Of the 76 imprinted genes listed in the Harwell database, 39 (51%) are paternally expressed. Assuming a 51% probability of paternal expression for each of the retrogene candidates, it is highly unlikely that all 11 would share this property by chance (*p* < 1 × 10^−3^).

**Figure 1:**
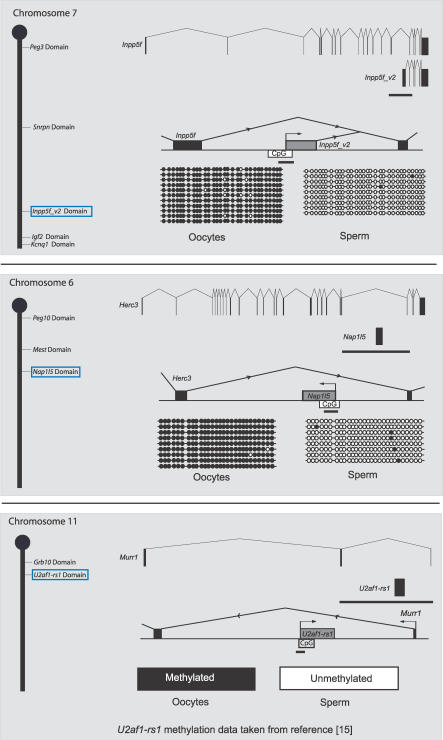
The Genomic Environment and Germ-Line Methylation Status of Three Imprinted Retrogenes The chromosome maps on the left-hand side show the position of each imprinted retrogene relative to other imprinted domains on the same chromosome. For each of the three loci, the top right-hand section shows the exonic structure and splicing pattern, the middle section shows the intron within which the retrogene is situated, and the bottom section shows the methylation status in oocytes and sperm, as determined by bisulphite sequencing. Circles on horizontal lines depict CpG dinucleotides on individual strands of genomic DNA. Filled circles represent methylated CpGs and open circles are unmethylated CpGs. The horizontal bar underneath each section marks the extent of the region below to depict scale. For *U2af1-rs1,* the methylation data is a summary of previously published results [15].

We hypothesized that retrogene insertions might attract differential methylation in the germ-line and hence play a role in the formation of imprinted domains during evolution. However, the imprinting of four retrogenes situated within the *Snrpn* imprinted cluster on mouse Chromosome 7 is controlled by a gDMR situated over 1 Mb from the retroposed sequences [18], and a similar situation has been reported for the *Dio3* and *Rtl1* genes on Chromosome 12 [19]. Rather than establishing new gDMRs, some retrogenes may acquire the pre-existing imprint status of their integration site [14]. For this reason, we excluded putative retrogenes that were located within larger imprinted clusters, where known gDMRs do not overlap retrogene exons (references in Table 1), leaving three genes (Table 2). Interestingly, all three are situated within introns of RefSeq-annotated multi-exonic host genes. Of the remaining eight, none are situated within introns of RefSeq genes. *U2af1-rs1* and *Nap1l5* are transcribed from the opposite strand to their host transcripts [13,20], whereas *Inpp5f_v2* is transcribed in the same orientation (Figure 1) [21]. At the *Inpp5f_v2* locus, transcripts containing the retroposed sequence are spliced onto downstream exons of *Inpp5f,* forming a chimeric gene [10,22].

**Table 2:**
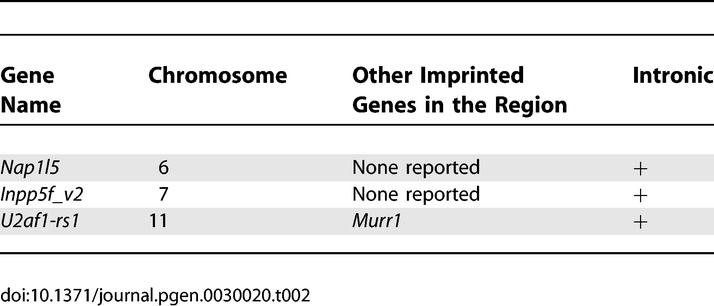
Murine Retrogenes Associated with gDMRs

### Methylation Analysis

The *Inpp5f_v2* and *Nap1l5* promoters are known to be methylated on the maternally derived allele in somatic tissues [20,21], but no gDMR had previously been identified at either of these imprinted loci. The methylation status of the CpG islands overlapping the two retrogene promoters was assessed by sequencing bisulphite-modified DNA from ovulated oocytes and mature sperm. Both regions are heavily methylated in female, but not male gametes (Figure 1). The *U2af1-rs1* promoter had previously been shown to undergo methylation specifically during oogenesis (Figure 1) [15]. The finding that the *U2af1-rs1, Nap1l5,* and *Inpp5f_v2* retrogenes all overlap gDMRs suggests that the inserted sequences are specifically targeted for methylation in the maternal germ-line.

### The Origins of Imprinted Retrogenes

**Figure 2:**
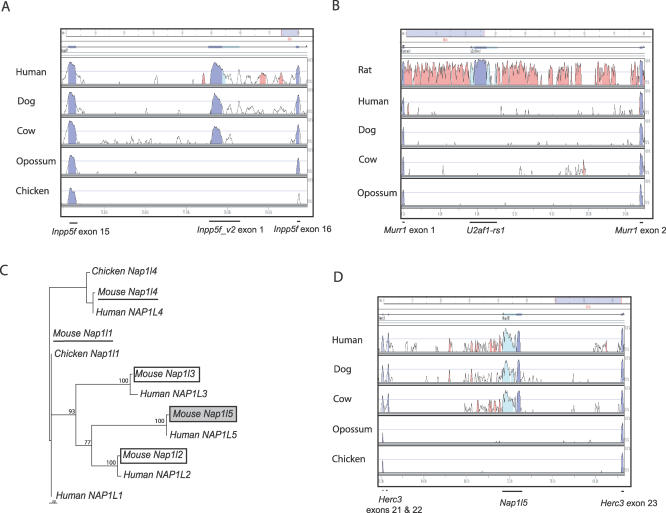
Multi-Species Comparative Sequence Analysis of Introns Containing Retrogenes and gDMRs in the Mouse Genome The genomic DNA sequence of the entire host gene was used to generate VISTA plots using mouse as the base genome. (A) *Inpp5f*/*Inpp5f_v2* and (B) *Murr1*/*U2af1-rs1.* Conserved sequences corresponding to coding exonic regions in the mouse are shaded purple, noncoding exons are light-blue, and conserved nontranscribed regions are pink. The position of exonic mouse sequences is indicated at the bottom of each plot. Presence or absence of the retroposed sequence in each species can be used to determine the approximate point in the mammalian radiation at which each retrogene originated. (C) Maximum likelihood (ML) tree showing members of the *Nap1l* family in mouse, human, and chicken. The alignment from which this tree was generated can be found in Figure S1. The imprinted mouse gene is within a shaded box, the two X-linked monoexonic paralogues are within open boxes, and the two autosomal and multi-exonic members are underlined. 100 trial bootstrap resampling scores are given for nodes relevant to the chromosomal origin of *Nap1l5*. (D) Multi-species comparative sequence analysis of *Herc3,* containing the imprinted *Nap1l5* retrogene. The VISTA plot is annotated in the same manner as (A and B).

To examine the retrotransposition events that generated these three genes in more detail, BLASTP searches were performed using the retrogene ORFs to identify all family members in mouse and human. Both *Inpp5f_v2* and *U2af1-rs1* belong to gene families consisting of only two closely related members, whereas the *Nap1l* family consists of five paralogues. The multi-exonic *Tmem114A* gene on the X chromosome is the only paralogue of the murine *Inpp5f_v2* ORF (also known as *Tmem114B*). The observation that the ORF of *Inpp5f_v2* is contained entirely within the first exon indicates a retrotransposition event originating from the *Tmem114A* gene on the X chromosome. Comparative sequence analysis using the genomic sequence of the *Inpp5f* gene in multiple species revealed the retrogene to be present in all eutherian mammals examined (Figure 2A). Absence of the retroposed sequence at the *Inpp5f* locus in the opossum genome demonstrates that this gene duplication event occurred after the marsupial divergence.

The X-linked, multi-exonic *U2af1-rs2* gene is the closest paralogue of the imprinted and monoexonic murine *U2af1-rs1* [13]. Applying the same logic as described for *Inpp5f_v2, U2af1-rs1* is the product of an X-to-autosome retrotransposition event [13]. A multi-species sequence comparison using the *Murr1* genomic sequence revealed that this event occurred in a common ancestor of mouse and rat, after the divergence of rodents and primates (Figure 2B). As previously reported [13,15], no orthologue of the murine *U2af1-rs1* sequence is present at the *MURR1* locus on human Chromosome 2.

The *Nap1l* gene family consists of five members, two of which are multi-exonic and possess orthologues in all vertebrates examined *(Nap1l1* and *Nap1l4).* Of the three monoexonic family members, the imprinted *Nap1l5* gene lies within an intron of *Herc3* on mouse Chromosome 6, whereas the *Nap1l2* and *Nap1l3* genes are situated on the X chromosome. The presence of three monoexonic paralogues makes their precise relationship complicated to determine, and so a maximum likelihood tree was generated using the region of the *Nap1* domain common to all five family members (Figure 2C). As the *Nap1l5* ORF is truncated and lacks regions of homology shared by all other family members (Figure S1), this gene cannot have acted as the source of *Nap1l2* or *Nap1l3.* Given this information, the imprinted paralogue is more likely to have originated from one of the two X-linked genes than from the autosomal *Nap1l1* or *Nap1l4* (supported by 93/100 bootstrap re-sampling trials; Figure 2C), implicating *Nap1l2* or *Nap1l3* as the likely source. At the *Nap1l5* locus, homology with other family members is limited to the transcribed sequence, and the flanking regions contain short target site duplications that are indicative of L1-mediated retrotransposition [23]. Based on these observations, the most likely origin of the *Nap1l5* gene is an X-to-autosome retrotransposition event, although the exact relationship between family members is less clear than for *Inpp5f_v2* and *U2af1-rs1.* Comparative sequence analysis using the *Herc3* genomic sequence reveals that this retrogene originated in a common ancestor of all eutherian mammals examined, but is absent in marsupials and nonmammalian vertebrate species (Figure 2D).

The promoter regions of the three retrogenes are associated with CpG islands in all species in which they are present. In contrast, CpG islands are absent in the orthologous intronic regions of genomes lacking the three retrogenes. The regions of CpG-rich sequence that undergo differential methylation in the germ-line therefore arose either during or shortly after the retrogene integration events. While it is possible to correlate the timing of the retroposon integrations with the origin of the corresponding CpG islands, the mechanism by which the CpG-rich sequences arose is unclear.

### Common Features of Imprinted Retrogenes

All three imprinted retrogenes that undergo differential methylation in the germ-line are situated within introns of multi-exonic genes and are likely to be derived from ancestral genes on the X chromosome. The X chromosome has generated a disproportionately large number of functional retrogenes over the course of mammalian evolution [24]. To contextualize our data, we collated a larger sample of mouse retrogenes that were assumed not to be imprinted. A detailed survey recently revealed 3,590 retrocopied gene duplicates in the human genome, 104 of which showed evidence of expression and originated in a common ancestor of rodents and primates. The 104 mouse retrocopies were manually annotated to identify those that had maintained an intact ORF and showed EST evidence of expression in the mouse genome (build v35, Text S1). A total of 74 mouse retrocopies fulfilled both of these criteria and are likely to represent bona fide mouse retrogenes (Dataset S2). Only one of the known imprinted retrogenes listed in Table 1 also features in this dataset *(Mkrn3),* suggesting that this sample does not contain a large proportion of the total number of retrogenes present in the mouse genome. Nonetheless, after excluding *Mkrn3,* the remaining 73 were deemed an adequate sample with which to compare the three gDMR-associated retrogenes. Approximately one in four (18/73) originated from the X chromosome, whereas approximately one in seven (10/73) were embedded within introns of RefSeq-annotated host genes. Although a formal statistical analysis is not possible with an *n* of 3, these data indicate that the properties of X-chromosome derivation and intronic location may be overrepresented among imprinted retrogenes overlapping gDMRs relative to their presumably nonimprinted counterparts.

### Identification of a Novel Imprinted Locus

**Figure 3:**
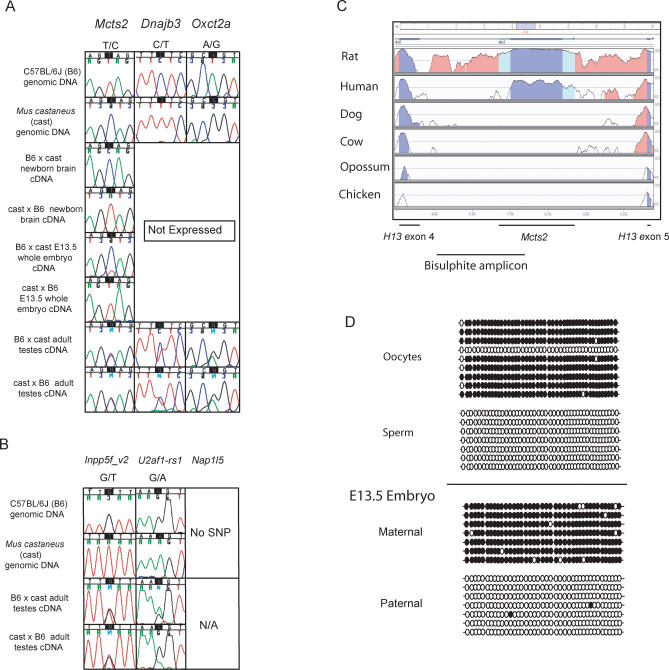
Identification of a Novel Imprinted Retrogene and gDMR (A) Allele-specific RT-PCR sequencing assays in inter-specific mouse hybrids. SNPs were identified between C57BL/6J (B6) and *Mus mus castaneus* (cast), such that the parental origin of the expressing allele(s) could be determined in F1 hybrids. The maternal allele is indicated first in the hybrid crosses. (B) Allele-specific RT-PCR sequencing assay for the *U2af1-rs1* and *Inpp5f_v2* genes in mouse testes. cDNA was prepared from whole testes. (C) Comparative analysis of the *H13* genomic sequence in multiple species, using mouse as the base genome. For clarity only the intron containing the imprinted murine retrogene is shown. Purple shading indicates coding exonic sequence, light-blue shading indicates noncoding exonic sequence, and pink shading indicates conserved nontranscribed sequence. Positions of mouse exons are shown as horizontal lines underneath the plot. (D) Methylation status of the *Mcts2* promoter region in germ cells and E13.5 embryo, determined by the sequencing of bisulphite-modified genomic DNA. Closed circles indicate methylated CpGs, open circles are unmethylated. E13.5 embryos were derived from B6 mothers and cast fathers, so the parental origin of each strand could be determined using a SNP within the PCR amplicon.

**Figure 4:**
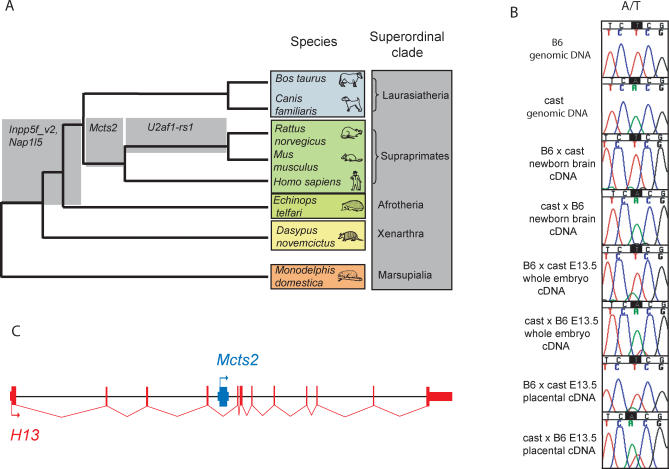
Evolutionary Tree for Placental Mammals, Using the Topology Determined in [54] (A) Based on the multi-species comparative sequence analysis in Figures 2 and 3, the approximate points in the mammalian radiation at which each of the four imprinted retrogenes originated are superimposed as grey boxes. The genome sequence of Dasypus novemcictus (armadillo) and Echinops telfari (tenrec) are currently only available in draft format and were therefore not used for the comparative analyses. (B) Allele-specific RT-PCR sequencing assays for *H13* in B6 × cast reciprocal F1 hybrids. The maternal allele is given first in the hybrid crosses. (C) Transcriptional overview of the *H13* locus. Protein-coding regions are shown as thick blocks, UTR regions as thin blocks, and introns as thin lines. Splice patterns are indicated. The paternally expressed *Mcts2* is shown in blue and the maternally expressed *H13* is in red. Arrows indicate the orientation of transcription.

Based on the data obtained from known imprinted loci, we hypothesized that X-derived retrogenes are more likely to be imprinted and associated with gDMRs than those derived from autosomes. In order to test this hypothesis, we selected all murine retrogenes from the sample of 73 (Dataset S2) that were situated within introns of known genes [25] and associated with CpG islands, regardless of their chromosomal origin. Only three retrogenes fulfilled both of these criteria, two of which were derived from parental genes on autosomes and one that was derived from the X chromosome (Table 3). Single nucleotide polymorphisms (SNPs) were identified between C57BL/6J (B6) and *Mus mus castaneus* (cast), and allele-specific RT-PCR sequencing assays were performed on cDNAs from reciprocal B6 × cast F1 hybrids. Primers were designed to specifically amplify the retrogene while avoiding amplification of other paralogous sequences, and specificity was confirmed by the alignment of sequence reads to the appropriate region of the mouse genome using the BLAT alignment tool [26]. The X-derived *Mcts2* was found to be expressed exclusively from the paternally derived allele in newborn brain, and a strong paternal allele bias was also seen in embryonic day (E) 13.5 embryo (Figure 4C). Expression of the two autosomally derived retrogenes, *Dnajb3* and *Oxct2a,* was not detectable by RT-PCR (35 cycles) in E13.5 embryo or placenta or neonatal brain (unpublished data). Although it was not possible to determine the imprinting status of these genes in somatic tissues, EST evidence suggested that they were both expressed exclusively in testes. The maternally and paternally derived alleles were expressed at approximately equal levels (Figure 3A), demonstrating that these two autosomally derived retrogenes do not undergo imprinting at their primary site of expression. We examined the imprinted expression of *Mcts2, U2af1-rs1,* and *Inpp5f_v2* in testes. All are expressed from both parental alleles in this tissue (Figure 3A and 3B), reflecting their unmethylated state in the male germ-line (Figures 1 and 3D). Although *Nap1l5* is expressed in testes, no SNP was identified within the transcribed region of this gene, and so imprinted expression could not be assessed.

**Table 3:**
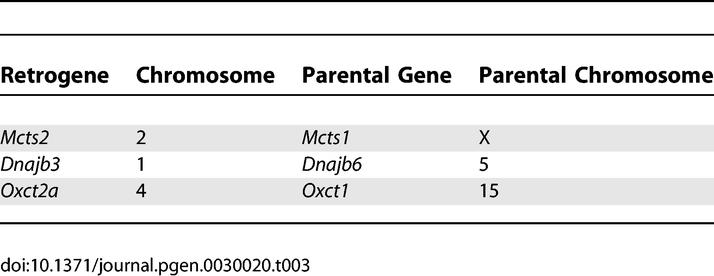
Murine Retrogenes Situated within Introns and Associated with CpG Islands

The X-derived retrogenes *U2af1-rs1, Nap1l5,* and *Inpp5f_v2* are all associated with gDMRs at CpG islands adjoining their promoters, which are in close proximity to the ORF-containing regions showing paralogy with the ancestral gene copy. To determine whether this was also the case at the *Mcts2* locus, the methylation status of the CpG island overlapping this promoter was examined by sequencing bisulphite-modified DNA from oocytes and sperm. Consistent with the results obtained for other intronic and X-derived retrogenes (Figure 1) [15], this region was predominantly methylated in oocytes but unmethylated in sperm (Figure 3D). Differential methylation of this region was also seen in E13.5 embryo (Figure 3D).

The *Mcts* gene family consists of two members in both mouse and human. The multi-exonic nature of the X-linked *Mcts1* confirms that the monoexonic *Mcts2* is an X-to-autosome retrogene, which lies within an intron of *H13.* Comparative sequence analysis was performed using the genomic sequence of *H13* in multiple species (Figure 3C). Although the retrogene is present in primates and rodents, it is absent in the genome of both dog and cow. *Mcts2* therefore originated in the supraprimate clade (synonymous with Euarchontoglires, including rodents and primates), after the laurasiatherian divergence (including canines and ruminants; Figure 4A).

### Imprinting of the Signal Peptide Peptidase *(H13)* Gene

Imprinted genes often occur in clusters, and individual gDMR sequences can control the imprinting of multiple neighbouring transcripts [27]. This raised the possibility that the gDMR at the *Mcts2* promoter could also control the imprinting of the more ancient *H13* gene within which it lies. Primers were designed to amplify exons 3 to 13, spanning the intron of *H13* within which the *Mcts2* gDMR is situated. Expression is exclusively from the maternally derived allele in newborn brain (Figure 4B), in contrast to the paternally expressed retrogene (Figure 3A). Although the maternally derived allele of *H13* is preferentially expressed in E13.5 embryo and placenta, the paternally derived allele is also active in these tissues (Figure 4B).

### Conservation of Imprinting in Human

**Figure 5:**
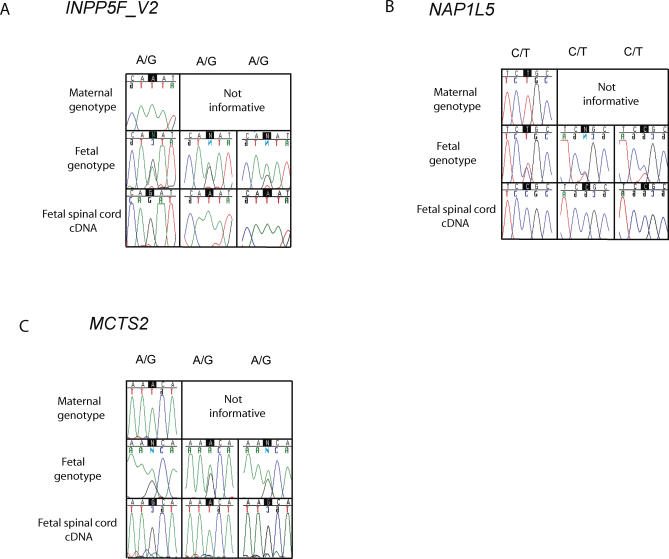
Allele-Specific RT-PCR Sequencing Assays for the Human *INPP5F_V2* (A), *NAP1L5* (B), and *MCTS2* (C) Transcripts The maternal and fetal genotype was determined for each family. Where mother and fetus were both heterozygous, the parental origin of the single expressing fetal allele could not be determined (“not informative”). For all three genes, the first family shows paternal-allele-specific expression in fetal spinal cord. In each case, the remaining two families exhibited monoallelic expression.

The retrotransposition events that generated the murine *Nap1l5, Inpp5f_v2,* and *Mcts2* genes occurred prior to the divergence of rodents and primates (Figure 4A), and the human orthologues are situated on Chromosomes 4 *(NAP1L5),* 10 *(INPP5F_V2),* and 20 *(MCTS2),* respectively. The imprinting status of these three genes had not been previously assessed. To address this, allele-specific assays were performed in fetal spinal cord cDNA with matched maternal DNA (Figure 5). SNPs were identified in fetal genomic DNA for each gene and the maternal genotype was determined. Where the mother and fetus were both heterozygous (“noninformative” families), the parental origin of the single expressing allele of an imprinted gene could not be determined. One informative family was obtained for each gene, and in every case expression was exclusively from the paternally derived allele in the fetus (Figure 5A–5C). Monoallelic expression was confirmed in two additional noninformative families. For every gene, monoallelic expression was observed in all tissues in which expression was detected, which included fetal brain, heart, and tongue (unpublished data).

## Discussion

From a systematic screen of known imprinted genes in the mouse, we identified three retrogenes that are closely associated with gDMRs. The observation that all three were embedded within introns and likely to be derived from parental genes on the X chromosome led to the discovery of a novel gDMR, which is associated with a previously undescribed cluster of imprinted transcripts. Other sequence-based studies of imprinted regions have identified some interesting associations, notably a correlation with direct repeat sequences [28] and a paucity of short interspersed elements at imprinted promoters [29]. In contrast to previous studies, the sequence features identified in this report have proved powerful enough to identify a novel imprinted locus, making *Mcts2*/*H13* the first such locus to be identified solely on the basis of bioinformatic data.

The ORF of *Mcts2* encodes a 181 amino acid protein that contains a PUA domain, putatively involved in RNA binding. Both the mouse and human ORFs show >90% sequence identity with the X-linked paralogue *Mcts1/MCTS1* (malignant T-cell amplified sequence 1), which acts as a positive regulator of cyclin-dependent cell-cycle progression [30]. Human lymphoid cells overexpressing *MCTS1* show a markedly reduced doubling time [31], and the gene is upregulated in several lymphoma cell lines [32]. *MCTS2* lies within a region of Chromosome 20q11 that is frequently amplified in a variety of cancers [33,34]. The identification of a functional paralogue of a known oncogene within this critical region may have implications for the pathogenesis associated with 20q amplification.

*H13* codes for signal peptide peptidase, an intra-membrane aspartic protease with homology to presenilin-like proteins [35]. This locus was first identified four decades ago, due to its role as a histocompatibility antigen causing tissue-incompatibility between inbred strains of laboratory mice [36,37]. Several other histocompatibility antigens are encoded by the mitochondrial genome [37–39] and are therefore subject to maternal transmission via a distinct mechanism to *H13.* While the shielding of fetal antigens from the maternal immune system is an attractive hypothesis to explain *H13* imprinting, relaxation of imprinted expression in the placenta argues against this theory.

Oocyte-derived methylation at the *Mcts2* promoter region is likely to be the primary epigenetic mark at the *H13* locus. The resulting paternal-allele-specific expression of the *Mcts2* retrogene may interfere with the transcription of *H13* in *cis,* preventing the formation of full-length *H13* mRNAs on the paternal allele. Retrogene-mediated transcriptional interference has also been suggested to account for the imprinting of the *Murr1* gene on mouse Chromosome 11 [40]. The fact that gDMRs overlap exonic sequences at *Mcts2, U2af1-rs1, Nap1l5,* and *Inpp5f_v2* suggests that the retrotransposed elements are integral to the imprinting mechanism at each locus. However, these data do not prove a causal role for the retrogene integrations in the evolution of imprinting at these loci, as the possibility of a pre-existing imprinted state cannot be excluded.

### Mechanistic Significance of Imprinted Retrogene Properties

Retrogenes that share the properties of X-derivation, intronic location, and association with a CpG island are rare in the mouse genome (one out of 74, Dataset S2); although there are several reasons to believe that additional examples could exist. Firstly, the dataset of retrocopied sequences published by Vinckenbosch et al. focused on the human genome [10]; therefore, only mouse retrogenes that originated in a common ancestor of rodents and primates were examined in this report. Genes acquired more recently in the rodent lineage (e.g., *U2af1-rs1*) would not have been detected, and so additional candidates might be revealed by an analysis focused on the mouse genome. Because of the stringent criteria that were necessarily applied, this study would also have omitted potential retrogenes that showed the greatest degree of similarity to monoexonic paralogues (e.g., *Nap1l5*).

Regardless of the total number of imprinted retrogenes that are present in the mammalian genome, the properties shared by each of the four examples identified in this report are likely to yield clues to the nature of the imprinting mechanism. All four gDMR-associated retrogenes are situated within introns of actively transcribed host genes. The fact that none are situated in intergenic regions suggests that transcription through the gDMR may be a necessary mechanistic component. Several other maternally methylated gDMRs are situated within introns *(Kcnq1ot1, Air, Nnat, Nespas, Gnas exon1A, Grb10),* indicating that this feature is common among elements that undergo methylation during oogenesis. Further work is required to determine the mechanistic significance of this property, but we speculate that transcription through the CpG island in germ cells may play a role.

The observation that all four gDMR-associated retrogenes have paralogues situated on the X chromosome suggests that this feature may also have mechanistic significance. Male and female germ cells differ in their sex chromosome constitution, and meiotic sex chromosome inactivation results in the transcriptional shutdown of X-linked genes during spermatogenesis. In contrast, X chromosomes are transcriptionally active during female meiotic prophase I [41], when maternal imprint marks are established [42]. It has been proposed by others that homology-dependent interactions between sex chromosomes and autosomes might underlie the sexually dimorphic patterns of DNA methylation that are established at imprinted loci during gametogenesis [43]. The idea that imprint establishment may involve interactions between homologous sequences is supported by the finding that mice carrying multiple copies of a *U2af1-rs1* transgene undergo aberrant methylation of the endogenous locus during spermatogenesis [44]. Homology-dependent transcriptional silencing of dispersed repeats has been reported in plants, funghi, diptera, and mammals [45–48], and dispersed Alu repeats in the primate genome undergo CpG methylation during female gametogenesis [49]. The Alu consensus sequence is <300 bp, suggesting that only relatively short regions of homology are required to induce these effects. The mechanistic similarities between retrotransposon silencing and genomic imprinting have been discussed for over a decade [4,5,43], and the discovery of four gDMRs associated with retrotransposed genes lends strong support to this proposed link.

### Retrogenes and Sexual Antagonism

The arguments above relate to the mechanisms by which imprinting is established at a locus, but do not extend to the processes by which natural selection may favor the spread of imprinted alleles within a population. In one model, it has been predicted that selection could favor the imprinting of genes that act in a sexually antagonistic manner, including those with roles in reproductive tissues such as the testes [50]. Several X-to-autosome retrogenes have acquired specific roles in the male germ-line [11,12], where they are thought to act as substitutes for their X-linked paralogues that are silenced by sex chromosome inactivation [51]. The expression pattern of *U2af1-rs1, Nap1l5, Inpp5f_v2,* and *Mcts2* appears to fit with this model, raising the possibility that imprinting could serve as a mechanism by which genes that have acquired specialized functions during spermatogenesis are silenced during female meiosis.

## Materials and Methods

### Bioinformatics.

Further details of the two screens by which Datasets S1 and S2 were generated are located in Text S1.

Protein sequences from the Nap1l family were aligned using CLUSTALW (http://www.ebi.ac.uk/clustalw), and the largest region showing clear homology between all aligned sequences (residues 66–137 of human NAP1L1, Figure S1) was used to generate a maximum likelihood tree using ProML within PHYLIP [52]. The tree topology generated by ProML was then tested for support from the alignment using bootstrap resampling analysis in GeneBee (http://www.genebee.msu.ru/services/phtree_reduced.html). mVISTA plots (http://genome.lbl.gov/vista/index.shtml) were generated using the following genome builds: mouse *(Mus musculus),* build 35; human *(Homo sapiens),* build 35; rat *(Rattus norvegicus),* Atlas v3.1; cow *(Bos taurus),* Btau_2.0; dog *(Canis familiaris),* v2.0; opossum *(Monodelphis domestica),* MonDom 2.0; and chicken *(Gallus gallus),* WASHUC1. The following genomic regions were compared: *Inpp5f:* mouse, Chr7:124707188–124793035; human, Chr10:121475663–121578648; cow, Chr26:25323898–25419575; dog, Chr28:32879108–32946035; opossum, scaffold_12290:987328–1016804; chicken, Chr6:29342310–29378953. *Murr1:* mouse, Chr11:22851733–22934290; rat, Chr14:103590704–103686780; human, Chr2:62044453–62274855; cow, Chr11:40264882–40438893; dog, Chr10:65037489–65209929; opossum, scaffold_13632:170442–420565. *Herc3:* mouse, Chr6:58800005–58872197; human, Chr4:89870824–89986864; cow, Chr6:19342489–19477683; dog, Chr32:14837425–14937677; opossum, scaffold_15026:3665556–3783621; chicken, Chr4:35520006–35557686. *H13:* mouse, Chr2:152410356–152447681; Rat: Chr3:142926299–142963222; human, Chr20:29565902–29621029; cow, Chr13:43262441–43302878; dog, Chr24:23996878–24039707; opossum, scaffold_13306:1536833–1561646; chicken, Chr20:9571122–9582505.

### Tissue sources.

Mouse: oocytes were derived from superovulated adult C57BL/6J females and sperm were dissected from the testes of adult males. Whole testes were used for the RT-PCR experiments, containing both somatic and germ-cell lineages. RNA was prepared by caesium chloride centrifugation, and oligo-dT primed cDNA was generated using the superscript first-strand kit (Invitrogen, http://www.invitrogen.com). Human: samples were collected under the guidelines of the Hammersmith and Queen Charlotte's and Chelsea Hospitals Trust Research Ethics Committee (Registration Number: 2005/6028). Informed consent was collected from all subjects.

### Bisulphite mutagenesis.

Oocytes were treated using a method adapted from Olek et al. [53]. Briefly, 50 oocytes were mixed with 10 μl molten LMP agarose and the mixture was solidified on ice and overlaid with cold mineral oil. After a 14-h incubation in lysis buffer (10 mM Tris-HCl [pH7.6], 10 mM EDTA, 1% SDS, 50 μg/ml proteinase K), agarose beads were washed for 3 × 15 min in TE before denaturing the DNA strands with 0.3 M NaOH for 2 × 15 min then 0.1 M NaOH for 1 × 10 min. NaOH solution was removed and replaced with 3.25 M Sodium MetaBisulphite (Sigma, http://www.sigmaaldrich.com) and 0.93 mM hydroquinone solution, which was overlaid with mineral oil prior to incubation at 55 °C for 5 h. Agarose beads were washed for 5 × 5 min in TE prior to incubation in 500 μl 0.2 M NaOH for 15 min at 37 °C then water for 2 × 10 min. The water was removed and the beads melted at 80 °C for 5 min and then aliquoted and used directly for PCR analysis. DNA from sperm and E13.5 embryos was treated essentially as above without encapsulation in agarose. Between two and five parallel amplifications were performed for each product.

### RT-PCR, bisulphite PCR, and sequencing.

All primers and cycling conditions that were used to amplify cDNA, genomic DNA, and bisulphite-modified DNA are detailed in Protocol S1. RT-PCR was performed for 30–35 cycles and −RT controls were run in parallel to control for genomic DNA contamination. Bisulphite PCR products were gel-purified using the QiaEXII (Qiagen, http://www1.qiagen.com) kit before cloning into the TOPO TA (Invitrogen) vector. Individual clones were sequenced using Big Dye v3.1 (ABI, http://www.abionline.com) sequencing technology. Between two and five independent amplifications were performed for each type of template, and strands from the same amplification that could not be distinguished on the basis of either epigenotype or unconverted non-CpG cytosines were excluded. All strands showed >95% conversion of non-CpG cytosines.

## Supporting Information

Dataset S1 Known Imprinted Genes in the Mouse (45 KB XLS)

Dataset S2 Mouse Retrogenes Originating Prior to the Rodent/Primate Divergence (34 KB XLS)

Text S1 Generation of Datasets S1 and S2
(31 KB DOC)

Figure S1 ORF Alignment of the Nap1l Protein Family (93 KB DOC)

Protocol S1 PCR Primers, Conditions, and SNPs (12 KB XLS)

### Accession Numbers

The GenBank (http://www.ncbi.nlm.nih.gov/Genbank) accession numbers for the genes discussed in this paper are *H13* (NM_010376), *INPP5F_V2* (AK091448), *MCTS2* (BC053868), *Mcts1* (also known as *Mct-1;* NM_026902), *Mcts2* (NM_025543), *NAP1L5* (NM_153757), *Nap1l2* (NM_008671), *Nap1l3* (NM_138742), *Nap1l5* (NM_021432), *Tmem114A* (BC028317), *Inpp5f_v2* (also known as *Tmem114B;* DQ648020), *U2af1-rs1* (NM_011663), and *U2af1-rs2* (NM_178754).

## Abbreviations

E: embryonic day
gDMR: germ-line differentially methylated region
ORF: open reading frame
SNP: single nucleotide polymorphism
