# Supplementary material for: A Screen for Retrotransposed Imprinted Genes Reveals an Association between X Chromosome Homology and Maternal Germ-Line Methylation
Source: PLoS Genet. 2007 Feb 9;3(2):e20. doi: 10.1371/journal.pgen.0030020 (PMC1796624; doi:10.1371/journal.pgen.0030020)
Supplement: Supplementary file 1 [file pgen.0030020.sd003.doc]

**Supplementary Methods**

**Generation of Supplementary Dataset 1**

The 76 imprinted genes currently listed on the Harwell imprinting website (<http://www.mgu.har.mrc.ac.uk/research/imprinting/imprin-viewdatagenes.html>) formed the basis of this dataset. Protein-coding capacity was inferred from the original research articles in which the imprinting of each gene was first described (listed on the following webpage: http://www.mgu.har.mrc.ac.uk/research/imprinting/imprin-generef.html). It should be noted that in some cases, translational capacity was inferred from ORF analysis without empirical verification. To identify imprinted genes likely to have been associated with sequence duplications, the BLASTZ alignment tool was utilised to identify those that showed homology with other regions of the mouse genome (self-alignments). Alignments were required to occur within the ORF region of protein coding imprinted genes, and were visualised using the self-chain track on the UCSC genome browser (Build 34, March 2005 http://genome.ucsc.edu). This track may not be available on more recent genome builds. The BLASTZ parameters necessary for a sequence to generate a self-alignment are described in the following references (Schwartz *et al* 2003, Kent *et al* 2003). Retrogene candidates were predicted to be protein coding, lack introns within their ORF and show BLASTZ self-alignments with their parent gene. The eleven genes that fulfilled all three of these criteria are highlighted, and listed separately in Table 1.

**Generation of Supplementary Dataset 2**

The 3590 human retrotransposed sequences identified by Vinckenbosch *et al* (2006) formed the basis of this dataset. The authors identified 1080 sequences with at least one overlapping EST, of which 104 originated in a common ancestor of rodents and primates. Manual annotation of this sample revealed 74 sequences with at least one overlapping EST in mouse genome build v35, and that had maintained an intact ORF by comparison with the parental gene. These 74 are likely to represent *bona fide* retrogenes in the mouse genome. It should be noted that the screening process would have missed retrogenes acquired in the last ~65 million years, after the divergence of rodents and primates. The one retrogene from this list known to undergo imprinting (*Mkrn3*) was also excluded, leaving 73 mouse retrogenes for which no evidence of imprinting had been reported previously. To allow each sequence to be traced back to the original manuscript in which it was identified (Vinckenbosch *et al* 2006), the ID in the left-hand column refers to the number assigned to the corresponding human orthologue described in the original report. Intronic is defined as initiating from within an intron of a known gene or Refseq gene on the UCSC genome browser ([http://genome.ucsc.edu](http://genome.ucsc.edu/)), and CpG islands were defined as genomic regions larger than 200bp that showed >50% G+C content, and an observed/expected CG dinucleotide ratio of > 0.6. The three retrogenes shaded pale blue are situated within introns and overlap CpG islands. The allele-specific expression of these three genes is examined in Figure 3 A.

# Supplementary References

Schwartz, S *et al*. (2003) Human-mouse alignments with BLASTZ. *Genome Research* 13: 103-107

Kent WJ *et al*. Evolution's cauldron: duplication, deletion, and rearrangement in the mouse and human genomes (2003) *PNAS* 100: 11484-11489.

Vinckenbosch, N. *et al*. Evolutionary Fate of Retroposed Gene Copies in the Human Genome. *PNAS* (2006) Feb 28; 103 (9):3220-5
