# Supplementary material for: A Screen for Retrotransposed Imprinted Genes Reveals an Association between X Chromosome Homology and Maternal Germ-Line Methylation
Source: PLoS Genet. 2007 Feb 9;3(2):e20. doi: 10.1371/journal.pgen.0030020 (PMC1796624; doi:10.1371/journal.pgen.0030020)
Supplement: Supplementary file 2 [file pgen.0030020.sg001.doc]

**Supplementary Figure 1**

```
                         10        20        30        40        50        60        70        80        90       100
                ....|....|....|....|....|....|....|....|....|....|....|....|....|....|....|....|....|....|....|....|
Human Nap1L1    MADIDN-KEQSELDQDLDDVEEVEE---EETGEETKLKARQLTVQMMQNPQILAALQERLDGLVETPTG-------------------------------
Mouse Nap1L1    MADIDN-KEQSELDQDLEDVEEVEE---EETGEETKIKARQLTVQMMQNPQILAALQERLDGLVDTPTG-------------------------------
Chicken Nap1L1  MADIDN-KEQSELDQDLDDVEEVEE---EETGEETKLKARQLTVQMMQNPQILAALQERLDGLVETPTG-------------------------------
Human Nap1L2    MAESENRKELSESSQEEAGNQIMVEGLGEHLERGEDAAAGLGDDGKCGEE-AAAGLGEEGENGEDTAAGS----------GEDGKKGGDTDEDSEADRPK
Mouse Nap1L2    MAESVDHKELSESNQEELGSQVMAEGPGESQDRSEGVSIEPGDGGQHGEETVAAGVGEEGK-GEEAAAGS----------GEDAGKCGGTDEDSDSDRPK
Human Nap1L3    MAEADF-KMVSEPVAHGVAEEEMAS---STSDSGEESDSSSSSSSTSDSSSSSSTSGSSSGSGSSSSSS-----------GSTSSRSRLYRKKRVPEPSR
Mouse Nap1L3    MAEADP-KMVTEPGAHGVAEEAMAS---TACDSGDESDSNSSSSTNSCSSSGSSSSGSSSSSSSSSSSSSSSSSSSSGSSGSSSNGSHLNRKKRVPEPSR
Human Nap1L4    MADH----SFS--DGVPSDSVEAAK---NASN-TEKLTD-----QVMQNPRVLAALQERLDNVPHTPSS-------------------------------
Mouse Nap1L4    MAEN----SLS--DGGPADSVEAAK---NASN-TEKLTD-----QVMQNPQVLAALQERLDNVSHTPSS-------------------------------
Chicken Nap1L4  MADN----SSSKAEDTASDSVEAAK---NASDKKEKLAD-----QVMQNPQVLAALQERLDNTALTPSS-------------------------------
Human Nap1L5    MADSEN-QGPAEPSQAAAAAEAAAEEVMAEGGAQGGDCDSAAGDPDSAAGQMAEEPQTPAENAPKPKND-------------------------------
Mouse Nap1L5    MADPEK-QGPAESR--------AEDEVMEG--AQGGE-DAATG--DSAAAPAAEEPQAPAENAPKPKKD-------------------------------
Dog Nap1L5      MADSEN-QGPAEPSQ---AAAEAAEEVMAEGGAQGGD-------SDSAAGPTAEEPQTPADNAPKPKND-------------------------------

                        110       120       130       140       150       160       170       180       190       200
                ....|....|....|....|....|....|....|....|....|....|....|....|....|....|....|....|....|....|....|....|
```

**Human Nap1L1** **----------YIESLPRVVKRRVNALKNLQVKCAQIEAKFYEEVHDLERKYAVLYQPLFDKRFEIINAIYEPTEEECEWKPD------------------**

```
Mouse Nap1L1    ----------YIESLPKVVKRRVNALKNLQVKCAQIEAKFYEEVHDLERKYAVLYQPLFDKRFEIINAIYEPTEEECEWKPD------------------
Chicken Nap1L1  ----------YIESLPRVVKRRVNALKNLQVKCAQIEAKFYEEVHDLERKYAVLYQPLFDKRFEIINAIYEPTEEECEWKPD------------------
Human Nap1L2    GLIGYVLDTDFVESLPVKVKYRVLALKKLQTRAANLESKFLREFHDIERKFAEMYQPLLEKRRQIINAIYEPTEEECEYKSDSEDCDDEEMCH-EEMYG-
Mouse Nap1L2    GLIGYLLDTDFVESLPVKVKCRVLALKKLQTRAAHLESKFLREFHDIERKFAEMYQPLLEKRRQIINAVYEPTEEECEYKSDCEDYFEEEMDEEEETNG-
Human Nap1L3    RARRAPLGTNFVDRLPQAVRNRVQALRNIQDECDKVDTLFLKAIHDLERKYAELNKPLYDRRFQIINAEYEPTEEECEWNSEDEEFSSDEEVQDNTPSEM
Mouse Nap1L3    RAQRRPSGKLFLDKLPQAVRNRVQALRNIQNECDKVDTLFLRAIHDLERKYAELNKPLYDKRFQIINAEYEPTEEECEWNSE-EEFSGDEEMQDDTPNEM
Human Nap1L4    ----------YIETLPKAVKRRINALKQLQVRCAHIEAKFYEEVHDLERKYAALYQPLFDKRREFITGDVEPTDAESEWHSENE----------------
Mouse Nap1L4    ----------YIETLPKAVKRRINALKQLQVRCAHIEAKFYEEVHDLERKYAALYQPLFDKRREFITGDVEPTDAESAWHSENE----------------
Chicken Nap1L4  ----------YIETLPKAVKRRIDALKQLQVKCAHIEAKFYEEVHDLERKYAALYQPLFDKRREFINGEAEPTDAESEWHSENE----------------
Human Nap1L5    ----------FIESLPNSVKCRVLALKKLQKRCDKIEAKFDKEFQALEKKYNDIYKPLLAKIQELTG-----EMEGCAWTLEGE----------------
Mouse Nap1L5    ----------FMESLPNSVKCRVLALKKLQKRCDKIEAKFDKEFQALEKKYNDIYKPLLAKIQELTG-----EMEGCAWTLEGE----------------
Dog Nap1L5      ----------FIESLPNSVKCRVLALKKLQKRCDKIEAKFDKEFQALEKKYNDIYKPLLAKIQELTG-----EMEGCAWTLEGEE---------------

                        210       220       230       240       250       260       270       280       290       300
                ....|....|....|....|....|....|....|....|....|....|....|....|....|....|....|....|....|....|....|....|
Human Nap1L1    ----------------------------------EEDEISEELKEKAKIE--------------------------DEKKDE------------------
Mouse Nap1L1    ----------------------------------EEDEVSEELKEKAKIE--------------------------DEKKDE------------------
Chicken Nap1L1  ----------------------------------EEDEISEELKEKAKIE--------------------------DEKKDE------------------
Human Nap1L2    ---------------------------------NEEGMVHEYVDEDDGYEDYYYDYAV------------------EEEEEEEEEDDIEATG--------
Mouse Nap1L2    ---------------------------------NEDGMVHEYVDEDDGYEDCYYDYD-------------------DEEEEEEEDDSAGATG--------
Human Nap1L3    PPLEGEEEEN---------------PKENPEVKAEEKEVPKEIPEVKDEEKEVP--------------KEIPEVKAEEKADSKDCMEATPEVKEDPKEVP
Mouse Nap1L3    PPLEGEEEEESCNEKAEVKEEGTHVPEEVPEAKVEEEEAPKETPEVKTEEKDIPKEGAEEKAEEQESSKEIPEVKGEEKADSTDCIDIAPEEKEDVKEVT
Human Nap1L4    ----------------------------------EEEKLAGDMKSKVVVT---------------------------EKAAATA----------------
Mouse Nap1L4    ----------------------------------EEDKLAGDMKNKVVIA---------------------------EKEAATV----------------
Chicken Nap1L4  ----------------------------------EEEKLAGDLKNVVVIE---------------------------EKAEA------------------
Human Nap1L5    ----------------------------------------------------------------------------------------------------
Mouse Nap1L5    ----------------------------------------------------------------------------------------------------
```

**Dog Nap1L5** **----------------------------------------------------------------------------------------------------**

```
                        310       320       330       340       350       360       370       380       390       400
                ....|....|....|....|....|....|....|....|....|....|....|....|....|....|....|....|....|....|....|....|
Human Nap1L1    -----------------------------------------------EKEDPKGIPEFWLTVFKNVDLLSDMVQEHDEPILKHLKDIKVKFSDAGQPMSF
Mouse Nap1L1    -----------------------------------------------EKEDPKGIPEFWLTVFKNVDLLSDMVQEHDEPILKHLKDIKVKFSDAGQPMSF
Chicken Nap1L1  -----------------------------------------------EKEDPKGIPEFWLTVFKNVDLLSDMVQEHDEPILKHLKDIKVKFSDAGQPMSF
Human Nap1L2    -------------------------------------------EENKEEEDPKGIPDFWLTVLKNVDTLTPLIKKYDEPILKLLTDIKVKLSDPGEPLSF
Mouse Nap1L2    -------------------------------------------GEEVNEEDPKGIPDFWLTVLKNVEALTPMIKKYDEPILKLLTDIKVKLSDPGEPLSF
Human Nap1L3    QVKADDKEQPKATEAKARAAVRETHKRVPEERLQDSVDLKRARKGKPKREDPKGIPDYWLIVLKNVDKLGPMIQKYDEPILKFLSDVSLKFSKPGQPVSY
Mouse Nap1L3    QANTENKDQP-TEEFTPRAPAREAQKRVPETRPEEGVNIKRARKGKPKKEDPKGIPDYWLTVLKNVDKLGPMIQKCDEPILKFLSDVSLKFSNPGQPIGY
Human Nap1L4    -----------------------------------------------EEPDPKGIPEFWFTIFRNVDMLSELVQEYDEPILKHLQDIKVKFSDPGQPMSF
Mouse Nap1L4    -----------------------------------------------EELNPKGIPEFWFTIFRNVDMLSELVQEYDEPILKHLQDIKVKFSDPGQPMSF
Chicken Nap1L4  -----------------------------------------------EEINVKGIPDFWFTIFRNVDMLSELVQEYDEPILKHLQDIKVKFSEPGQPMSF
Human Nap1L5    ----------------------------------------------------------------------------------------------------
Mouse Nap1L5    ----------------------------------------------------------------------------------------------------
Dog Nap1L5      ----------------------------------------------------------------------------------------------------

                        410       420       430       440       450       460       470       480       490       500
                ....|....|....|....|....|....|....|....|....|....|....|....|....|....|....|....|....|....|....|....|
Human Nap1L1    VLEFHFEPNEYFTNEVLTKTYRMRSEPDDSDPFSFDGPEIMGCTGCQIDWKKGKNVTLKTIKKKQKHKGRGTVRTVTKTVSNDSFFNFFAPPEVPESGD-
Mouse Nap1L1    VLEFHFEPNDYFTNEVLTKTYRMRSEPDDSDPFSFDGPEIMGCTGCQIDWKKGKNVTLKTIKKKQKHKGRGTVRTVTKTVSNDSFFNFFAPPEVPENGD-
Chicken Nap1L1  VLEFHFEPNEYFTNEVLTKTYRMRSEPDDSDPFSFDGPEIMGCTGCQIDWKKGKNVTLKTIKKKQKHKGRGTVRTVTKTVSNDSFFNFFAPPEVPESGD-
Human Nap1L2    TLEFHFKPNEYFKNELLTKTYVLKSKLAYYDPHPYRGTAIEYSTGCEIDWNEGKNVTLKTIKKKQKHRIWGTIRTVTEDFPKDSFFNFFSPHGITSNGR-
Mouse Nap1L2    TLEFHFKPNEYFKNELLTKTYVLKSKLACYDPHPYRGTAIEYATGCDIDWNEGKNVTLRTIKKKQRHRVWGTVRTVTEDFPKDSFFNFFSPHGISLNGG-
Human Nap1L3    TFEFHFLPNPYFRNEVLVKTYIIKAKPDHNDPFFSWGWEIEDCKGCKIDWRRGKDVTVTTTQSRTTATG--EIEIQPRVVPNASFFNFFSPPEIPMIGK-
Mouse Nap1L3    TFEFHFLPNPYFRNELLMKTYIIRSKPDHYDPFFAWGWEIEECKGCKIDWRRGKDVTVTTTRSRPGITG--EIEVQPRVVPNASFFNFFSPPEIPLIGK-
Human Nap1L4    VLEFHFEPNDYFTNSVLTKTYKMKSEPDKADPFSFEGPEIVDCDGCTIDWKKGKNVTVKTIKKKQKHKGRGTVRTITKQVPNESFFNFFNPLKASGDGES
Mouse Nap1L4    VLEFHFEPNDYFTNPVLTKTYKMKSEPDKADPFSFEGPEIVDCDGCTIDWKKGKNVTVKTIKKKQKHKGRGTVRTITKQVPNESFFNFFSPLKASGDGES
Chicken Nap1L4  SLEFPLGPNDYFPNTVLTKTYKMKSEPDKTDPFSFEGPEIVDCDGCTIDWKKGKNVTVKTIKKKQKHKGRGTVRTITKQVPNDSFFNFFNPIKVSGDGES
Human Nap1L5    ----------------------------------------------------------------------------------------------------
Mouse Nap1L5    ----------------------------------------------------------------------------------------------------
Dog Nap1L5      ----------------------------------------------------------------------------------------------------

                        510       520       530       540       550       560       570       580
                ....|....|....|....|....|....|....|....|....|....|....|....|....|....|....|....|....|..
Human Nap1L1    LDDDAEAILAADFEIGHFLRERIIPRSVLYFTGEAIEDDDD-DYDEEGEE--ADEEGEEEGDEENDPDYDPKKDQNPAECKQQ----
Mouse Nap1L1    LDDDAEAILAADFEIGHFLRERIIPRSVLYFTGEAIEDDDD-DYDEEGEE--ADEEGEEEGDEENDPDYDPKKDQNPAECKQQ----
Chicken Nap1L1  LDDDAEAILAADFEIGHFLRERIIPRSVLYFTGEAIEDDDD-DYDEEGEE--ADEEGEEEGDEENDPDYDPKKDQNPAECKQQ----
Human Nap1L2    -------DGNDDFLLGHNLRTYIIPRSVLFFSGDALESQQEGVVREVNDAIYDKIIYDNWMAAIEEVKACCKNLEALVEDIDR----
Mouse Nap1L2    -------VENDDFLLGHNLRTYIIPRSVLFFSGDALESQQEGVVREVNDEIYDKIIYDDWMAAIEEVKACCKNLEALVEDIDR----
Human Nap1L3    LEPREDAILDEDFEIGQILHDNVILKSIYYYTGEVN------------------GTYYQFGKHYGNKKYRK----------------
Mouse Nap1L3    LEPREDAILDEDFEIGQILHDNVILKSIYYFTGEIN------------------DPYYHDFRDYGNRKYYK----------------
Human Nap1L4    LDEDSEFTLASDFEIGHFFRERIVPRAVLYFTGEAIEDDDNFEEGEEGEE--EELEGDEEGEDEDDAEINPKV--------------
Mouse Nap1L4    LDEDSEFTLASDFEIGHFFRERIVPRAVLYFTGEAIEDDDNFEEGEEGEE--EELEGDEEGEDEDDADVNPKV--------------
Chicken Nap1L4  LDEDSEFTLAADFEIGHFFRERIVPRAVLYFTGEAIEDDDNFEEGEEGEE--EELEGEEEGEEEEDAESDPKV--------------
Human Nap1L5    -------------------------------------EEEEEEYEDDEEEG--EDEEEEEAAAEAAAGA---KHDDAHAEMPDDAKK
Mouse Nap1L5    -------------------------------------DDEDDEEEDDEEEE-----EEEEAAAGA-------------TGGPNFAKK
Dog Nap1L5      ------------------------------------DDDDEEEYED-EEEGEEEEEEEEEAAAEAAVEAAAAKDEGPHSAVPDDAKK
```

**Supplementary Figure Legend**

ORF alignment for the *Nap1l* family. The region common to all family members (yellow box) was used to generate the maximum likelihood tree in Figure 2 C. Note that the Nap1l5 ORF is truncated and lacks regions of homology shared by all other family members.
